# A new species of *Pseudosinella* Schäffer, 1897 (Collembola, Entomobryidae) from Altamira Caves (Cantabria, Spain)

**DOI:** 10.3897/zookeys.989.52361

**Published:** 2020-11-09

**Authors:** Enrique Baquero, Rafael Jordana, Lucía Labrada, Carlos G. Luque

**Affiliations:** 1 University of Navarra, Faculty of Sciences, Department of Environmental Biology, University Campus, 31080 Pamplona, Spain University of Navarra Pamplona Spain; 2 Impress Group Consulting S.L., P.O. Box 879, 39080 Santander, Cantabria, Spain Impress Group Consulting S.L. Santander Spain; 3 Coastal Interpretive Centre, Santander City Council, Barrio Monte-La Maruca, 56D, 39012 Santander, Cantabria, Spain Coastal Interpretive Centre Santander Spain

**Keywords:** cave dwelling fauna, chaetotaxy, northern Spain, *Pseudosinella altamirensis* sp. nov., taxonomy

## Abstract

This paper describes *Pseudosinella altamirensis***sp. nov.** from the Altamira Caves, municipal district of Santillana del Mar (Cantabria, Spain), and five other caves near the coast of Cantabria (northern Spain). Its taxonomic position is discussed and differences and similarities among morphologically and geographically close species are highlighted. The new species can be identified by its five eyes, the basal and small inner paired teeth on the claw, and the length of the uncrenulated part of the distal dens.

## Introduction

**Figure 1.**
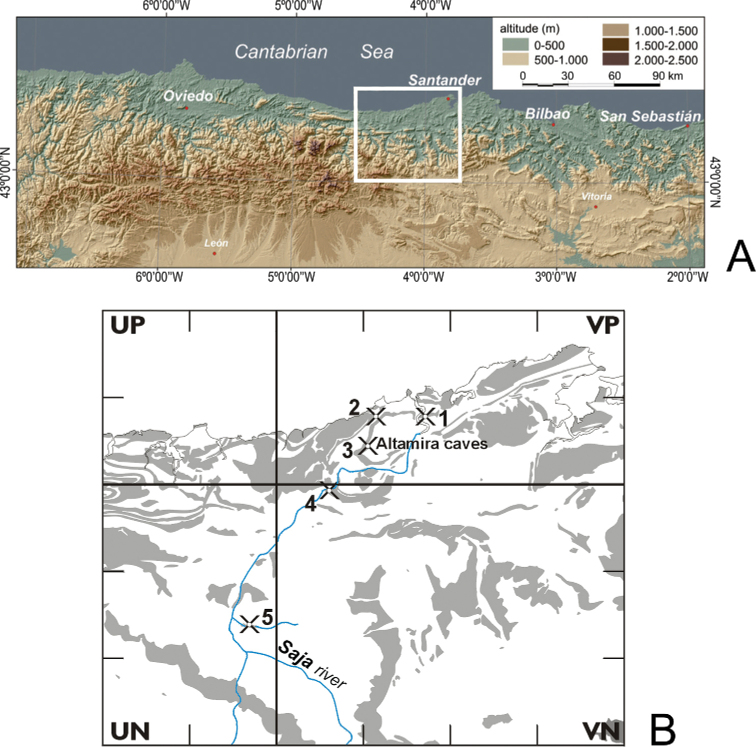
Location of the studied area. **A** Cantabrian Mountains (northern Spain) **B** UTM grid map with 10 km squares of the central region of the Cantabrian Mountain to show the outcrops of the rock systems (in grey) which contain limestone and geographical situation of the study area with the distribution of the cave-dwelling *Pseudosinella altamirensis* sp. nov.: **(1)** Cudón cave; **(2)** La Venta del Cuco cave; **(3)** Altamira and Stalactites caves; **(4)** Peña Caranceja cave; **(5)** Cobezo cave. Source: Spanish Geological Institute and Cartographic Service of the Cantabrian Government, scale 1:50 000.

During sampling work to increase the knowledge of the Collembolan cave fauna of the Cantabrian Mountains, we have captured a considerable number of undescribed species of Collembola. Some of these, mainly *Pseudosinella* Schäffer, 1897, are new species, and it is necessary to describe them. In this paper, a new species of the genus *Pseudosinella* is described, found in some limestone caves within the municipal districts of Miengo, Santillana del Mar, Reocín, and Cabuérniga (Fig. 1). A complete review of specimens collected in Altamira and other nearby caves (see Luque and Labrada 2016 for details) has shown that this new species was erroneously attributed to *Pseudosinella superoculata* Gisin & Gama, 1969 by Luque and Labrada. Dorsal macrochaetotaxy and other morphological characters have been used here for species identification. Christiansen et al. (1983) produced a catalogue of world *Pseudosinella* species and established a code for dorsal macrochaetotaxy characters. Christiansen et al. (1990) designed a computer-assisted identification key (Delta key) of the species of *Pseudosinella* with more characters. This electronic key is now available on the Web and regularly updated (Jordana et al. 2020). The new species was easily detected using this electronic key by the combination of chaetotaxy formula and other characters used in *Pseudosinella* species description.

## Materials and methods

### Abbreviations used in the description and figures:

**Abd** abdominal tergite

**accp** accessorial p-sensilla

**Ant** antennal segment

**a.s.l.** above sea level

**Mc** macrochaeta(e)

**mes** mesochaeta(e)

**mic** microchaeta(e)

**MZNA** Museum of Zoology, University of Navarra

**pse** pseudopore

**Th** thoracic tergite

### Terminology

Dorsal head and body chaetotaxy follows Gisin (1965, 1967a, b), Szeptycki (1979), and Soto-Adames (2010). Equivalence between the notation proposed by Gisin and the AMS system *sensu*Soto-Adames 2010 is given in Table 1.

**Table 1.** Equivalence table of Gisin (1965, 1967a, 1967b) notation formulae for chaetotaxy to the modern notation of Soto-Adames 2010 (AMS) as presented in the figures.

	Dorsal Mc formula																	Additional sensilla		Abd II formula				
	Head							Th			Abd													
Character from Gisin	R_0_	R_1_	R_2_	S	T	P	/	Th II	Th III	/	Abd	A2	A3	Abd IV	+	Abd IV	/	s	Character from Gisin	p	a	b	q_1_	q_2_
Possible status	0–1	0–1	0–1	0–1	0–1	0–1		0–2	0–2		0	0–2	0	0–1		0–3		0–1	Possible status	- –p	a–A	b–B	q_1_–Q_1_	q_2_–Q_2_
Actual status	mic–Mc	mic–Mc	mic–Mc	mic–Mc	mic–Mc	mic–Mc		mic–Mc number						mic–Mc		mic–Mc number		presence–absence	Meaning	0–mic	mic–Mc	mic–Mc	mic–Mc	mic–Mc
Chaeta AMS system notation	A_0_	A_1_	A_2_	M	S_2_ or S_4_	Pa_5_		m or p series			m or p series			C_1_		row B		s	Chaeta AMS system notation	a_2p_	a_2_	m_3_	m_3e_	p_4_

**Table 2.** Diagnostic characters (C1–C22) for the separation of the species of *Pseudosinella* that share the dorsal macrochaetotaxy formula with the new species.

Species	C1	C2	C3	C4	C5	C6	C7	C8	C9	C10	C11	C12	C13	C14	C15	C16	C17	C18	C19	C20	C21	C22	D
*P. alpina*	3*	1*	2	2	4	2	2	2	1*	1	3	1*	2	3*	3	5–7*	1	2.4*	U	32–36*	1.7–2.0	3*	9
*P. astronomica*	U	1*	4	2	4	2	2	2	1*	1	3	1*	1	3*	U	U	1	1.85*	U	60*	1.4*	U	7
*P. christiani*	1*	0*	2–4	2	4	2	2	2	1*	1	3	1*	1	3*	U	U	1	1.5*	U	35*	1.5*	1	8
*P. goughi*	1*	6–7*	2–4	2	4	2	2	2	2	1	2*	2	1	3*	U	U	1	1.9*	25–30	–	1.7–1.8*	U	6
*P. mucronata*	1*	5–6	2	2	4	2	2	2	1*	1	3	1*	1	2*	U	U	1	2*	U	60*	1.8–2*	U	7
*P. suboculata*	2	5–6	4	2	4	2	2	2	2	2*	3	2	1–2	2*	U	U	3*	2.5*	35	70*	1.7–1.8*	2	6
*P. superoculata*	1*	6*	4	2	4	2	2	2	2	1	3	1*	1–2	3*	3*	9–10	1	2.3*	30	45–50	2.1–2.3	2	6
*P. thibaudi*	U	2*	4	2	4	2	2	4*	1*	1	3	1–2	1	U	2	2–3*	2*	1.3*	U	70*	1.3–1.4*	U	8
*P. vandeli relicta*	1*	0*	4	2	4	2	2	2	1*	1	3	1*	2	3*	U	U	1	U	U	30–37	1.5*	2	6
*P. altamirensis* sp. nov.	2	5	2–4	2	3–4	2	2–4	2	2	1	3	2	1–2	1	2	7–12	1	3.1	35	40	2.16	2	–

Legend. **C1**: apical organ of third antennal segment: (1) peg or rod-like, (2) expanded. **C2**: number of eyes per side. **C3**: M1 < ventral labial chaeta > shape: (2) smooth Mc, (3) ciliated mic or mes, (4) ciliated Mc. **C4**: M2 < ventral labial chaeta > shape: (2) smooth Mc. **C5**: R < ventral labial chaeta > shape: (3) ciliated mic or mes, (4) ciliated Mc. **C6**: E < ventral labial chaeta > shape: (2) smooth Mc. **C7**: L1 < ventral labial chaeta > shape: (2) smooth Mc, (4) ciliated Mc. **C8**: L2 < ventral labial chaeta > shape: (2) smooth Mc. **C9**: Abd IV supplementary seta s: (1) absent, (2) present. **C10**: tenent hair shape: (1) acuminate, (2) clavate. **C11**: claw total teeth number. **C12**: claw wing tooth: (1) absent, (2) present. **C13**: empodium appendage wing tooth: (1) smooth, (2) minute. **C14**: empodium appendage shape: (1) acuminate, (2) truncate, (3) basally swollen. **C15**: inner chaeta on manubrial plate, number. **C16**: outer chaeta on manubrial plate, number. **C17**: habitat: (1) cave, (2) surface, (3) both cave and surface. **C18**: maximum length, in mm. **C19**: distance distal paired claw tooth from base/total claw, %. **C20**: distance distal unpaired claw tooth from base/total claw, %. **C21**: Antennae/head ratio. **C22**: differentiated inner chaeta on hind tibiotarsus: (1) unclear or absent, (2) clear, acuminate, (3) clear, truncate or clavate. **D**: accumulated differences. “*”, difference with *P. altamirensis* sp. nov. “U”, unknown. “-“, not applicable.

The characters defined by Christiansen et al. (1990) for *Pseudosinella* and those used by Christiansen (2013) and Jordana et al. (2020) in the Delta key have been used for identification. Some characters proposed by Mateos (2008) and Winkler and Mateos (2018) have also been considered.

### Study area

The Saja River catchment is in the central sector of the Cantabrian mountain range and flows to the Cantabrian Sea after following a course of 67 km in which it descends approximately 1700 m in altitude. It follows a practically rectilinear south-north course in its middle and upper sections, and in its lower one it flows in a west-east direction, which changes to a south-north direction in its final reach at the mouth of the San Martín de la Arena estuary between the municipalities of Miengo and Suances (Fig. 1). This course coincides with the main fracturing and folding lines which characterise the area. In the two municipalities which compose the middle course (Ruente and Cabuérniga), a dismantling of Cretaceous sandstones and clays has taken place, promoting the rising to the surface of Jurassic carbonate materials (Fig. 1). Among the few cavities known over these Jurassic limestones are the caves of Poyo and Cobezo (also the caves located in the Saja-Besaya Natural Park) and the flooded Fuentona de Ruente cave. The first Creaceous limestone outcrops are in the largest municipalities (Cabezón de la Sal, Reocín, Santillana, and Miengo), which are located along the middle-lower course of the Saja River. In the geologic context, this coastal area is distinguished by the abundance of lower to upper Cretaceous carbonates, which favours karst development. Among the abundant explored cavities in these limestone rocks are two (the caves of Peña Caranceja and La Venta del Cuco) that harbor the new species described here. Cudón cave, which has prehistoric paintings, is near the town of the same name at the opening of the Saja River that forms the San Martín de la Arena estuary; Cudón cave is approximately 9 km from Altamira Cave (Fig. 1).

**Figure 2.**
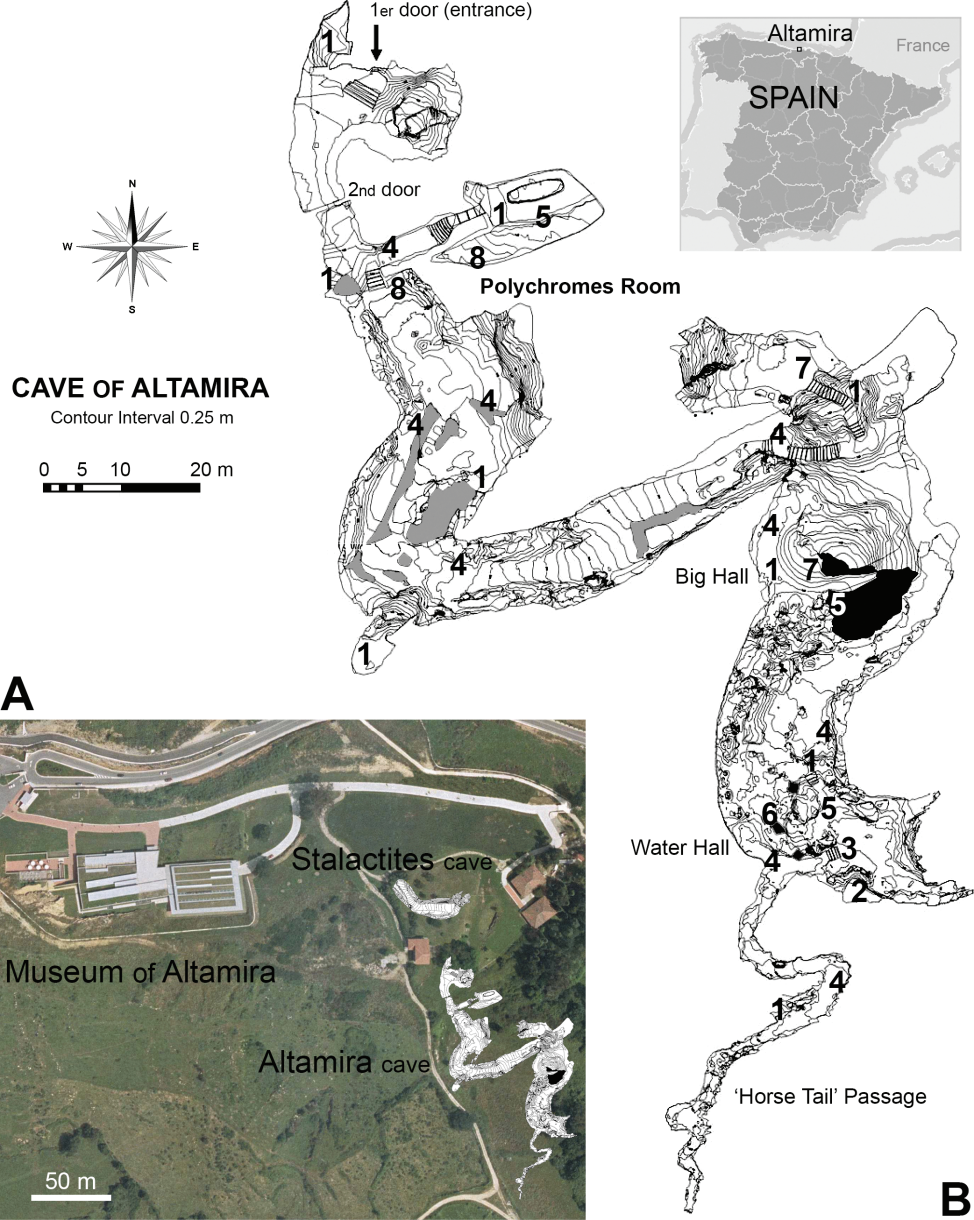
Geographical location of Altamira Caves (Cantabria, Spain). **A** location and ground plan of the Altamira Caves and its museum **B** location of the *Pseudosinella altamirensis* sp. nov. sampling sites along Altamira Cave (August 2000): **(1)** areas where soil contained mouldy rat or bat faeces; **(2)** areas where soil contained a rat cadaver (*Rattus rattus*); **(3)** areas where soil contained a bat cadaver (*Rhinolophus hipossideros*); **(4)** areas where walls had insect cadavers with visible fungal; **(5)** areas where water drips or flows towards the cave (hygropetric habitats); **(6)** areas where water had flooded the surface with small rim stone dams or gours; **(7)** areas with high clay soil content in the water; **(8)** areas of continuous monitoring of radon. Plan of the caves with its location courtesy of the National Museum and Research Centre of Altamira.

The Altamira Cave is in the central region of Cantabria (northern Spain), in one of the limestone hills that surround the small valley in Santillana village. On the whole, the geological and structural characterisation of Altamira Cave (270 m in length) indicates the evolution of a karstic complex from the first prehistoric human occupation until present. It is one of the many caves in the upper vadose area of the tabular polygenic karstic system that developed on Cretaceous calcarenite limestones (Sánchez et al. 2007). This area is known as Santa Olaja, although it is also known by the name of Planes (Santillana del Mar), where the medieval chronicles place the site of the “Sant[a] Illana” monastery (Luque and Labrada 2016). The small plain that extends over the Altamira Cave has an elevated position (159 m a.s.l.) below the Mount Santa Olaja hill range (168 m a.s.l.), and is protected between the mounts of Vispieres (226 m a.s.l.) and Cildad (287 m a.s.l.). This area separates the Saja River catchment from the Santillana physiographic basin (66 m a.s.l.). Altamira Cave, lying 4 km from the sea, is little more than 2 km from the nearby Saja River. It is situated on a topographical high point (152 m a.s.l.) and is only 3–22 m (averaging 8 m) below the surface. Having a length of 270 m, the cave features a main passage 2–12 m high, and 6–20 m wide (Fig. 2). It was discovered in 1868; exploration began in 1875, but it was not until 1879 when the first paintings, one of the first to be cataloged as Palaeolithic, were discovered by Marcelino Sanz de Sautuola (Lasheras 2009). Regarding the cave biology, Ignacio Bolívar was the first entomologist to explore the Altamira Cave in July 1883 (Luque and Labrada 2014, 2016). Since then, entomological visits to the cave have been rather sporadic. For example, in the early 20^th^ century, Charles Alluaud (28 June 1913) and Cándido Bolívar (28 August 1915) explored this cave (Luque and Labrada 2014, 2016). On 7 May 1924, the cave and its surrounding area was protected by a resolution of the Government of Spain. Since 1985, the cave and its paintings have been included in the UNESCO list of World Heritage Sites (SC-85/Conf. 008/9 1985).

Furthermore, a small cave with stalactites was discovered in the summer of 1928 when stone was being quarried out for the construction of a road to Altamira Cave. It is purely of geological interest, with beautiful stalactites and stalagmites, as no cave art has been found within. In October 1935, Hans Jürgen Stammer was the first entomologist that explored the Stalactites Cave (Luque and Labrada 2016). This cave also hosts the new species described here.

### Specimen collection and measurement

The specimens were obtained by direct capture. Additionally, baited pitfall traps were used in the parts of the cave that were considered favourable for the presence of fauna (Fig. 2) (see Luque and Labrada 2016 for details). The specimens were captured using a manual aspirator and then preserved in vials containing 70% ethyl alcohol. Each vial was labelled with the following data: collection site, coordinates, date of capture, name of the organisation, and person involved in the capture. The specimens were mounted in Hoyer medium, and optical observations were made under an Olympus BX51-TF microscope with a multiviewing system and phase contrast, and an Olympus BX50-F4 microscope with differential interference contrast (DIC). For the measurements, a U-DA drawing attachment UIS (Universal Infinity System) and a scale calibrated with a slide by Graticules Ltd (1 mm divided in 100 parts) was used. For electron microscopy, three specimens were fixed with 4% (v/v) glutaraldehyde in 0.1 M cacodylate buffer (pH 7.3) for 48 h, then stored for 24 h in a 0.25 M sucrose buffer containing 0.1 M cacodylate, and dehydrated using an ethanol series followed by critical-point drying in CO_2_, mounted on aluminium SEM stubs, and coated in Argon atmosphere with 16 nm of gold in an Emitech K550 sputter-coater. SEM observations and photographs were made with a Zeiss DSM 940 A.

## Taxonomy

### Class Collembola Lubbock, 1873

**Order Entomobryomorpha Börner, 1913**

**Family Entomobryidae Schäffer, 1896**

**Subfamilia Lepidocyrtinae Wahlgren, 1906**

**Genus *Pseudosinella* Schäffer, 1897**

#### Pseudosinella altamirensis sp. nov.

Taxon classificationAnimaliaEntomobryomorphaEntomobryidae

2CFF569E-776D-5B8D-8B6E-1FE4FB66B87B

http://zoobank.org/2f1163c5-c7d2-43e7-93a7-b7d30c2f11d0

**Figure 3.**
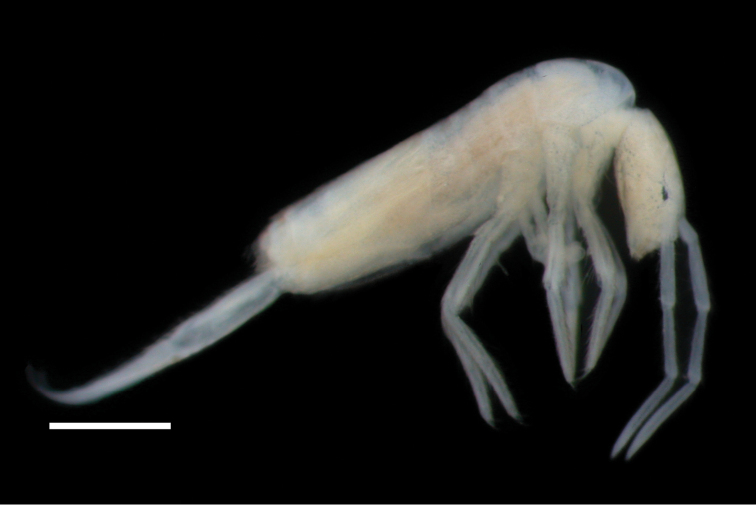
*Pseudosinella altamirensis* sp. nov. Habitus. Scale bar: 0.5 mm.

**Figure 4.**
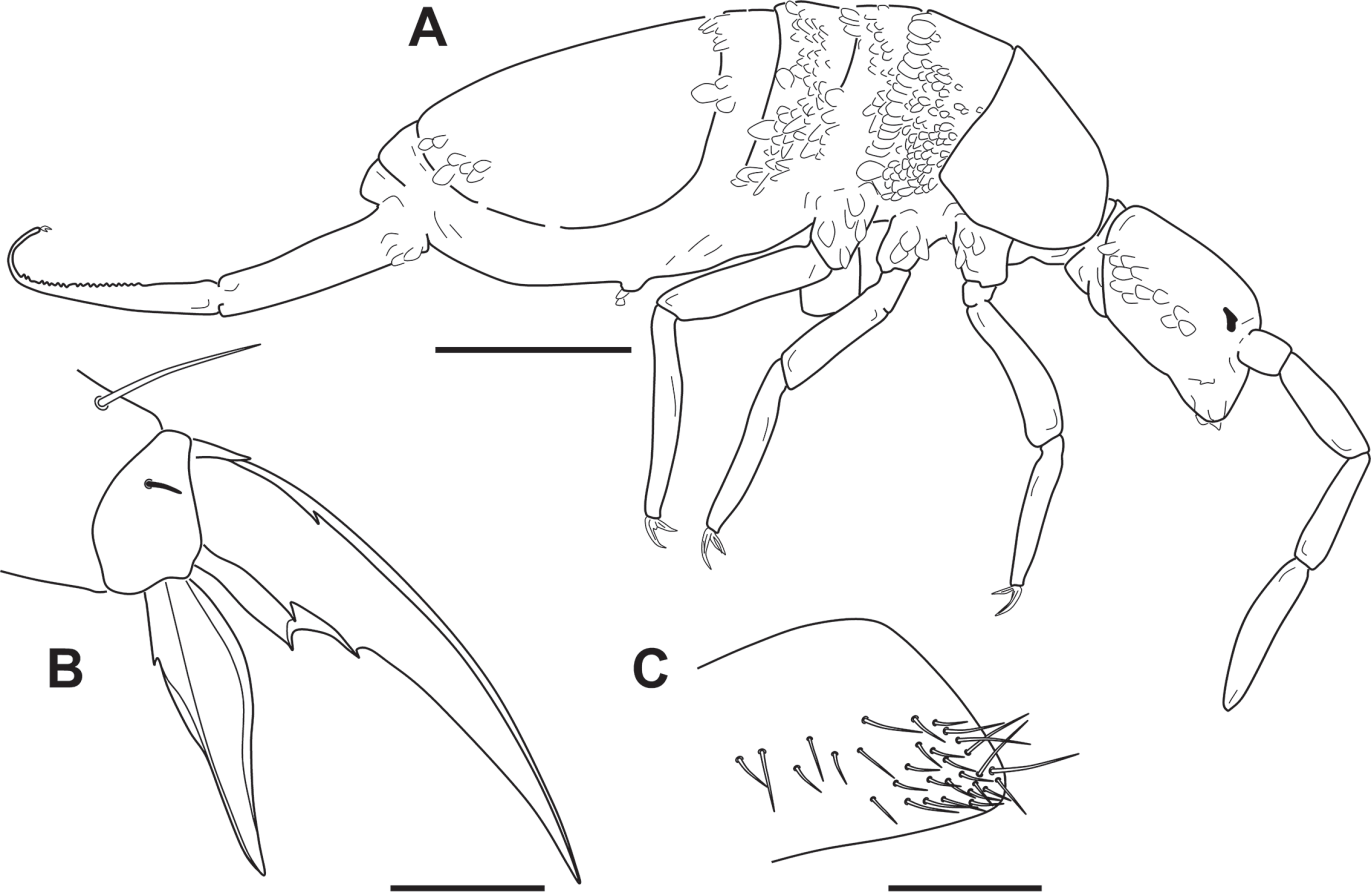
*Pseudosinella altamirensis* sp. nov. **A** habitus **B** distal part of tibiotarsus III and claw complex **C** trochanteral organ. Scale bars: 0.5 mm (**A**); 0.02 mm (**B, C**).

**Figure 5.**
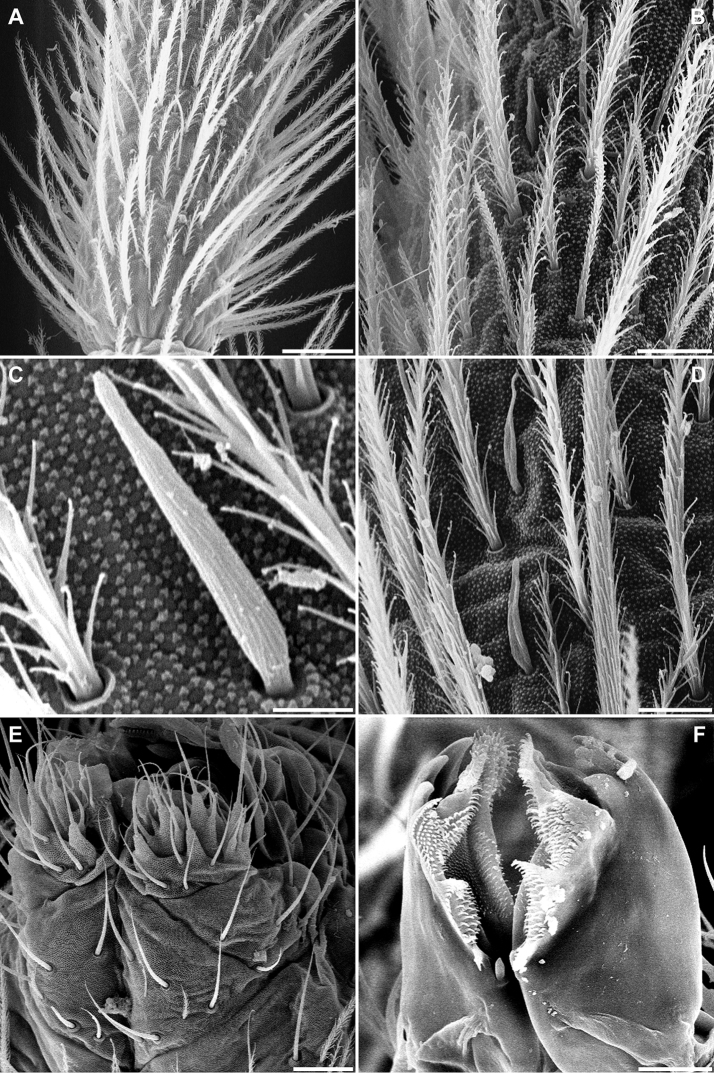
*Pseudosinella altamirensis* sp. nov. **A** proximal half of Ant IV **B** detail of Ant IV showing normal chaetae and different types of sensilla: leaf shaped, smooth, with small cilia **C** detail at high magnification of the small sensilla narrowed at tip found on Ant II–IV **D** two leaf-shaped sensilla on Ant IV **E** labium and labral palps **F** maxillae. Scale bars: 0.02 mm (**A, E**); 0.007 mm (**B**); 0.002 mm (**C**); 0.006 mm (**D**); 0.009 mm (**F**).

**Figure 6.**
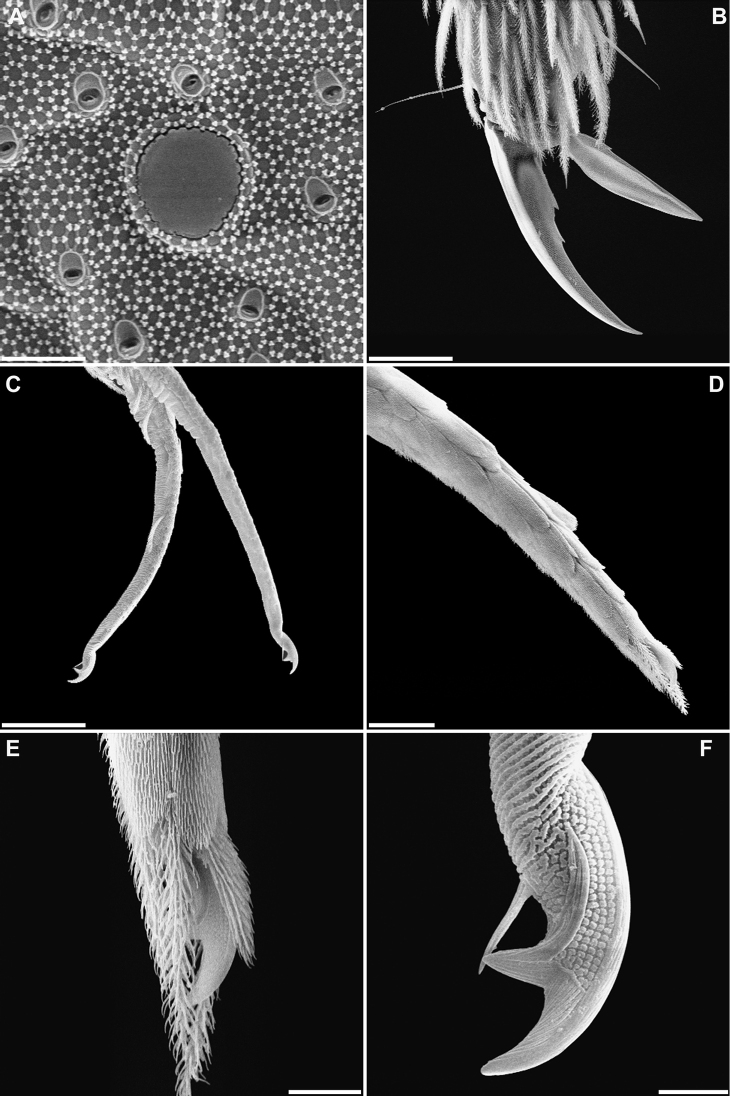
*Pseudosinella altamirensis* sp. nov. **A**Th II pseudopore **B** claw and empodium appendage of leg III **C** distal par of dens and mucro **D** tip of dens and mucro, not visible by the covering scales of terminal dens **E** mucro partially covered by scales and chaetae **F** mucro and mucronal spine. Scale bars: 0.004 mm (**A, F**); 0.02 mm (**B, D**); 0.04 mm (**C**); 0.007 mm (**E**).

**Figure 7.**
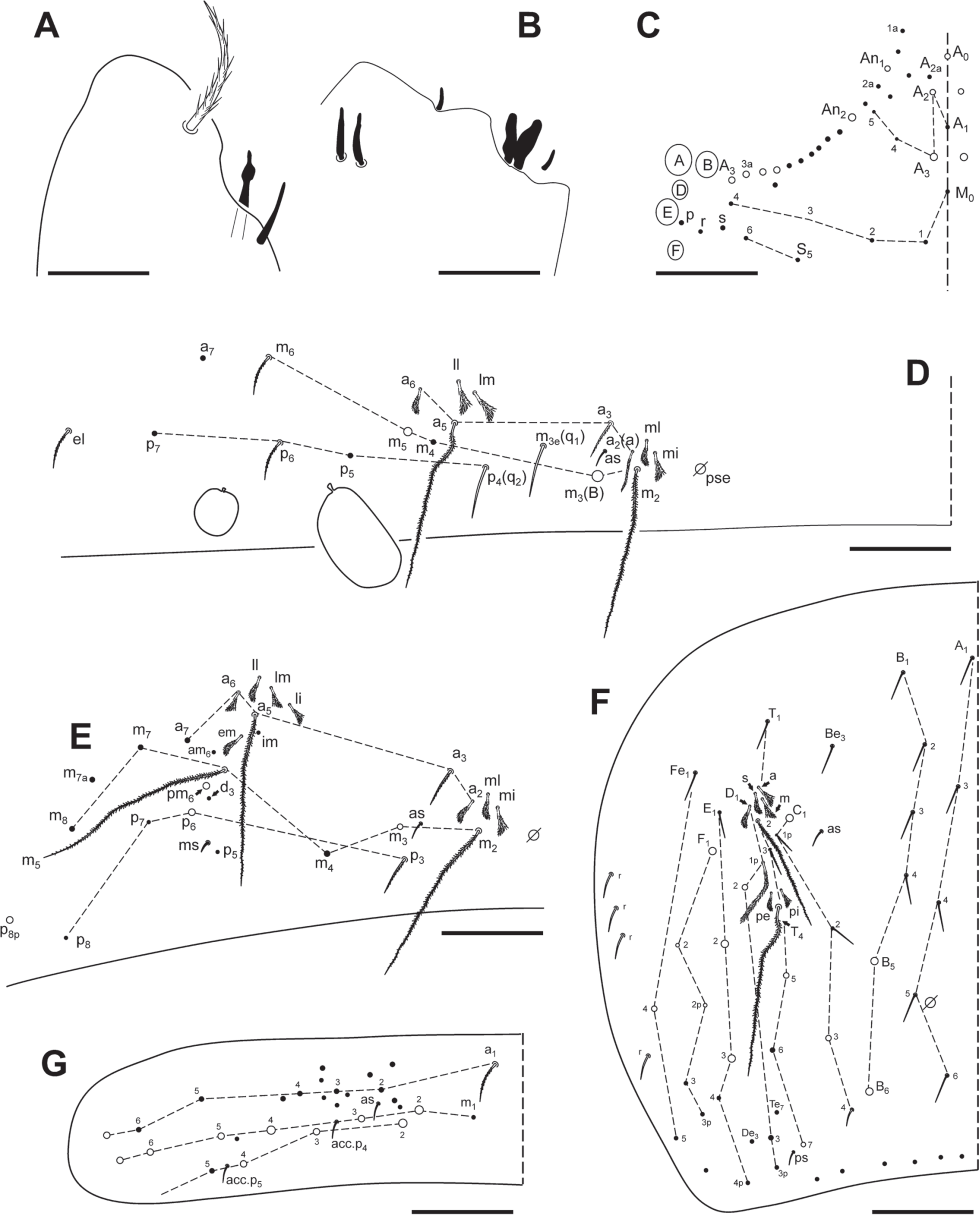
*Pseudosinella altamirensis* sp. nov. **A** antennae, tip of Ant IV **B** antennae, Ant III sensory organ **C** head, dorsal chaetotaxy; Abdomen dorsal chaetotaxy: **D** abd II **E** abd III **F** abd IV **G** abd V. White dots, Mc (size of the socket proportional to reality); black dots, mic. Scale bars: 0.01 mm (**A**); 0.02 mm (**B**); 0.05 mm (**C–E**); 0.1 mm (**F, G**).

Figs 3 , 4 , 5 , 6 , 7

##### Type material.

***Holotype***: Spain • ♀; Cantabria, municipal district of Santillana del Mar, **Altamira Cave**, Sala de Polícromos (Polychromes Room), National Museum and Research Centre of Altamira; 43°22.61'N, 4°7.18'W; 148 m a.s.l.; 29 Aug. 2000; C. Glez.-Luque leg.; slide labelled “MZNA -Altamira 6d-01”. ***Paratypes***: • 49 specimens on ethyl alcohol and 3 specimens mounted on SEM stubs; Cueva de las Estalactitas (Stalactites cave); 43°22.64'N, 4°7.21'W; 148 m a.s.l.; 29 Aug. 2000; C. Glez.-Luque leg.; slides MZNA -Altamira 6d-02 to 05 • 3 specimens on slides and 25 in ethyl alcohol; Polychromes Room; 43°22.61'N, 4°7.18'W; 148 m a.s.l.; 24 Mar. 2008; Cesáreo Saiz leg.; slides MZNA -Altamira01-01 and MZNA -Altamira01-02; deposited at the Museum of Zoology, University of Navarra, Pamplona, Spain (MZNA ).

##### Other material.

Spain – **Cantabria** • 2 specimens on slides and 14 on ethyl alcohol; La Venta del Cuco cave, Ubiarco, Santillana del Mar; 43°24.28'N, 4°6.35'W; 145 m a.s.l.; 13 Sep. 1995; C. Glez.-Luque leg.; slides MZNA -Luque Coll. 13d-01 and 02 • 1 specimen on slide and 1 in ethyl alcohol; Cudón cave, Cudón, Miengo; 43°24.94'N, 4°0.74'W; 22 m a.s.l.; 14 Sep. 1995; C. Glez.-Luque leg.; slide MZNA -Luque Coll. 36d • 1 specimen on slide and 3 in ethyl alcohol; Peña Caranceja or La Peñona cave, Barcenaciones, Reocín; 43°20.33'N, 4°9.45'W; 125 m a.s.l.; 7 Oct. 2000; C. Glez.-Luque leg.; slide MZNA -Luque Coll. 3d • 2 specimens on slide and three in ethyl alcohol; Cobezo, Cobeján or Perro cave, Viaña, Cabuérniga; 43°11.61'N, 4°16.52'W; 360 m a.s.l.; 15 Jun. 1996; C. Glez.-Luque leg.; slide MZNA -Luque Coll. 7d.

##### Diagnosis.

5 + 5 ocelli. Antennae moderately long. Ant III sense organ with two paddle- or leaf-shaped and two additional sensilla. Area not ringed of dentes nearly five times the length of mucro. Claw elongate, with two paired basal teeth; tenent hair acuminate. Reduced formula: R_0_R_1_R_2_000/00/0101+2/s, -aBq_1_q_2_, M_1_m_2_R*el_1_l_2_ (* 1/3 to 2/3 of M; sometimes M_1_ smooth and L_2_ ciliated, and usually asymmetrically).

##### Description.

***Habitus*** (Figs 3, 4A). Body length up to 3.1 mm (holotype: 2.3 mm). Colour: blue pigment laterally on body from Th II to Abd IV, but extended to dorsal part in Th II, coxae I–III, first third of femur III, laterally on head and vertex and Ant I–III. Abd IV paler. Eyes and a spot on central vertex strongly pigmented.

***Head***. With five eyes (ABDEF or ABCDF; C and F smaller, almost imperceptible in some specimens). Intraocular chaetae p, r, and s present. Only A_0_, A_2_, A_3_, An_1_, An_2_, An_3a_, and An_3_ as Mc (Fig. 7C). Ratio antenna/cephalic diagonal 1.64–2.16. Antennal segments I/II/III/IV ratios 1/2–2.7/2.3–2.7/3.5–4.1. Ant IV proximal area in Fig. 5A; apical vesicle absent (Fig. 7A); in this segment there are up to four special leaf-shaped sensilla on the distal third, two of them aligned, and at least five other different types of sensilla (some described by Beruete et al. 2002): smooth and cylindrical, some with short fringes, clearly different from the normal chaetae (Fig. 5B); smooth, shorter and narrowed in its distal third (Fig. 5C); and leaf-shaped, similar to the sensorial chaetae ‘s’ of sensory organ of Ant III (Fig. 5D). Ant III sense organ with a peculiar configuration: two paddle-shaped sensilla (individually encased in a pit and more or less one above the other), another two similar ones but in a dorso-internal position, and two small, rounded, spiny guard sensilla on both sides of the first one (Fig. 7B). Apical region of the Ant II–III with pseudopore in internal-ventral position, far from chaetae line. Antennae without scales. Prelabral chaetae ciliated; labral row a, m, and p all smooth (distal row (a) on papillae). Formula of the labial base M_1_m_2_Rel_1_l_2_; M_1_ sometimes smooth, asymmetrical in some specimens; R ciliated, 1/3 to 1/2 length of the neighbouring smooth Mc m_2_; l_1_ occasionally ciliated; the remaining chaetae smooth, but with minute fringes or ciliation seen only in SEM (Fig. 5E). Bifurcate maxillary palp with three smooth sublobal chaetae. Labial papilla (l.p.) E with finger-shaped process just reaching the base of apical appendage. Maxilla in Fig. 5F.

***Body***. Th II without Mc; pseudopore of this tergite in Fig. 6A. Th III without Mc. Abd II: chaetae a, q_1_ and q_2_ as ciliated mic, chaeta B as broad ciliated Mc (Fig. 7D). Abd III chaetotaxy shown in Fig. 7E. Accessory chaeta ‘s’ in the anterior trichobothrial complex of Abd IV present. Medial chaeta B_5_ below the level of the trichobothrium T_4_. Pseudopore between B_5_ and B_6_. Legs scales only on coxae. Trochanteral organ with ca 30 chaetae (Fig. 4C). Remaining chaetae clearly visible on all legs, acuminate and largest on leg III. Differentiated supraempodial inner chaeta on hind tibiotarsus well differentiated and acuminate. Dorsal tibiotarsal tenent hairs acuminate, 0.3 times the length of inner margin of claw. Claw with only three internal teeth: dental plate occupying 35% of the basal internal edge, with the basal paired teeth of different sizes (posterior one larger and slightly more distal than anterior); unpaired tooth well developed, approximately 40% from base; lateral tooth, anterior, less frequently posterior, present in some specimens on leg I and in basal positions; dorsal tooth basal, observed only in one specimen at SEM. *Empodium* appendage acuminate, basally swollen, externally smooth, with a minute tooth in some specimens (Figs 4B, 6B). Retinaculum with 4 + 4 teeth and one ciliated chaeta. Ventral tube without scales; lateral flap with a maximum of eight smooth and six ciliated chaetae. Manubrium and dens with scales only ventrally (anteriorly); two internal and 7–12 external chaetae related to two distal pseudopores of manubrial plate; area not ringed of dentes nearly five times the length of mucro; mucro with distal tooth longer than the anteapical; basal spine reaching the tip of distal tooth (Fig. 6C–F). Chaetotaxy from head to Abd V illustrated in Fig. 7C–G.

##### Biology.

This species is always found over organic matter accumulation. In Cudón cave, it has been found over the residue of rotten and wet wood; in the other caves it was found over old, mouldy rat and bat faeces and generally in insect cadavers with visible fungi. Although this species reaches to the dark zone of the caves near very wet areas, it has been found in deep zones on walls, roofs, and soils where the environmental humidity is near the saturation point (Fig. 2). The species appears to be restricted to the karst systems associated with the Saja River and within the municipal districts of Miengo, Santillana del Mar, Reocín, and Cabuérniga (Luque and Labrada 2016) (Fig. 1).

##### Etymology.

The name is toponymical and refers to the type locality, the Altamira Cave, one of the most important Palaeolithic art sites in Europe.

## Discussion

According to the dorsal macrochaetotaxy (R000/00/0101+2), the presence of chaeta ‘s’ in the anterior trichobothrial complex of the Abd IV, the Abd II chaetotaxy (-aBq_1_q_2_), and the formula of the labial base (M_1_m_2_Rel_1_l_2_; M_1_ or m_1_; L_1_o l_1_), this species is similar to *Pseudosinella goughi* Gisin & Gama, 1972, *P. suboculata* Bonet, 1931, and *P. superoculata* (with more constant labial base formula: M_1_m_2_Rel_1_l_2_ except in the case of *P. goughi* with M_1_ or m_1_). Nevertheless, these three species have six eyes (A, B, C, D, E, and F). *Pseudosinella suboculata* can have five eyes, but its dorsal tibiotarsal tenent hair is clavate and its claw is clearly different, with longer paired teeth, impaired tooth distal (more than 60% from base of inner claw), and the empodium appendage not basally swollen. *Pseudosinella superoculata* has the paired teeth of the inner claw at the same level, claw approximately 30 % longer than in the new species, manubrial plate with three chaetae (two in one specimen) internal to pseudopore, and sensorial chaetae ‘s’ of sensory organ of Ant III rod-like (after Gisin and Gama 1969 and observed also in some specimens from the Cantabria and Navarra caves). *Pseudosinella goughi* exceptionally has up to seven eyes and only two internal teeth on claw. In addition, the four special leaf-shaped sensilla present on Ant IV in the new species separate it from its most similar species. With the same dorsal macrochaetotaxy, but without chaeta ‘s’ on Abd II, there are six species: *P. alpina* Gisin, 1950 (one eye); *P. astronomica* Gisin & Gama, 1970 (one eye); *P. christiani* Stomp, 1986 (without eyes); *P. mucronata* Gouze & Deharveng, 1987 (five or six); *P. thibaudi* Stomp, 1977 (two eyes); and *P. vandeli relicta* Gisin, 1964 (no eyes). The differences between these nine species and the new species are shown in Table 1.

## Supplementary Material

XML Treatment for Pseudosinella altamirensis
